# Measurement Method for Height-Independent Vegetation Indices Based on an Active Light Source

**DOI:** 10.3390/s20071830

**Published:** 2020-03-25

**Authors:** Yongqian Ding, Yizhuo Jiang, Hongfeng Yu, Chuanlei Yang, Xueni Wu, Guoxiang Sun, Xiuqing Fu, Xianglin Dou

**Affiliations:** 1 College of Engineering, Nanjing Agricultural University, Nanjing 210031, China; 2017112013@njau.edu.cn (Y.J.); hongfengyu@njau.edu.cn (H.Y.); 2018812043@njau.edu.cn (C.Y.); 2018112027@njau.edu.cn (X.W.); sguoxiang@njau.edu.cn (G.S.); fuxiuqing@njau.edu.cn (X.F.); 1993042@njau.edu.cn (X.D.); 2 National Engineering and Technology Center for Information Agriculture, Nanjing 210095, China; 3 Jiangsu Key Laboratory for Intelligent Agriculture Equipment, Nanjing 210031, China

**Keywords:** active light source, vegetation indices, reflectance spectrometer, measurement method

## Abstract

A coefficient *C_W_*, which was defined as the ratio of NIR (near infrared) to the red reflected spectral response of the spectrometer, with a standard whiteboard as the measuring object, was introduced to establish a method for calculating height-independent vegetation indices (VIs). Two criteria for designing the spectrometer based on an active light source were proposed to keep *C_W_* constant. A designed spectrometer, which was equipped with an active light source, adopting 730 and 810 nm as the central wavelength of detection wavebands, was used to test the Normalized Difference Vegetation Index (NDVI) and Ratio Vegetation Index (RVI) in wheat fields with two nitrogen application rate levels (NARLs). Twenty test points were selected in each kind of field. Five measuring heights (65, 75, 85, 95, and 105 cm) were set for each test point. The mean and standard deviation of the coefficient of variation (CV) for NDVI in each test point were 3.85% and 1.39% respectively, the corresponding results for RVI were 2.93% and 1.09%. ANOVA showed the measured VIs possessed a significant ability to discriminate the NARLs and had no obvious correlation with the measurement heights. The experimental results verified the feasibility and validity of the method for measuring height-independent VIs.

## 1. Introduction

The spectral reflectance of the crop canopy can provide information about crop growth. Using the spectral reflectance of the crop canopy can effectively realize dynamic monitoring of crop growth characteristics and nutritional status. Moreover, nitrogen content is often the most important information for indicating crop nutritional status [1,2,3,4,5]. The non-destructive detection of crop nitrogen content is an important application of spectral information detection technology in the field of agriculture. By using this technology, combined with the diagnosis model of crop nitrogen nutrition, we can quickly obtain the nutrition information of crop growth inside a specific field size, which is conducive to the generation of the decision-making prescription of the nitrogen application rate and to the realization of variable rate fertilization. It is practically significant for the promotion of precision agriculture technology [6,7,8,9,10].

According to the amount of detection wavebands, the current reflectance spectrometers can be divided into super-spectral, hyper-spectral, multi-spectral and specific-spectral types of spectrometers [11,12]. The first three kinds of reflectance spectrometers are usually more complicated and expensive, and are mainly used in aerial remote sensing or laboratory research to explore the quantitative relationship between crop growth and vegetation indices (Vis) with specific wavebands. Both super-spectral and hyper-spectral spectrometers have much higher spectral resolution than multi-spectral spectrometers and can produce continuous spectral measurement results. Super-spectral spectrometers have the highest spectral resolution, which can generate hundreds or even thousands of detection wavebands in the measurement wavelength range (generally 350–2500 nm), and hyper-spectral spectrometers usually include dozens or, at most, hundreds of detection wavebands, while the multi-spectral spectrometers only possess a few or a dozen. Super-spectral spectrometers mostly adopt the active scanning mode as the imaging mode and the Michelson double beam interference light splitting mode for light splitting. Correspondingly, hyper-spectral spectrometers usually use the optical imaging mode and optical grating light splitting mode, while multi-spectral spectrometers adopt optical imaging technology and linear gradient optical filter for light splitting [13,14,15]. The specific-spectral spectrometers are for special purposes based on growth diagnosis models in which two detection wavebands in the visible and near-infrared wavelength range are involved. Moreover, the optical filters are usually used to ensure the bandwidth of the detection wavebands [16,17,18,19,20,21]. Due to the characteristics of portable carrying, low cost and high suitability, specific-spectral spectrometers are widely used in the real-time diagnosis of crop growth and can be divided into passive and active types according to the property of the light source.

The earlier versions (in the 1990s) of specific-spectral spectrometers (such as GreenSeeker by NTech Industries Inc., and N-sensor by Hydro Agri Gmbh) are all belong to the category of passive light source instruments. This type of spectrometer uses sunlight as the detection light source. The biggest limitation is that the measurement results are easily affected by the variations in intensity of sunlight, sun elevation angle, atmospheric conditions, etc. In particular, they cannot be used in low illuminance environments (such as cloudy days, early morning or the late afternoon on sunny days) [22,23]. Bajwa et al. used a portable dual-band reflectance spectrometer (Analytical Spectral Devices, developed by Boulder Co.) to measure the spectral reflectance of a rice canopy. They found that it was appropriate to use the device at 10:00 a.m.–15:00 p.m. in clear weather to decrease the influence of the change in the sun’s elevation angle on the measurement results [1]. Peteinatos’s review also mentioned that, with weed monitoring sensors, the measured results were easily affected by the change of ambient light environments because of changes in the cloud layer, the atmospheric variances and sun elevation angle [24]. Yang et al. carried out a reliability analysis of Cropsense (developed by Beijing Research Center for Information Technology in Agriculture, China) and SRS_NDVI (a commercial product of Decagon Co., USA). The experimental results showed that the suitable measurement condition was related to the reliability of the spectrometers. If the intensity of sunlight was greater than 5.2 klux, the influence of light intensity and sun elevation angle was not significant, and the reliability of the spectrometers decreased obviously under other conditions [25].

In order to overcome the limitation in application of the spectrometers based on passive light sources, spectrometers based on active light sources began to appear in the late 1990s. This type of spectrometer is characterized by its own light source with specific wavebands. The light source is illuminated in a high frequency interval mode. The purpose is to make the frequency of the spectrometer response signal caused by the active light obviously higher than that caused by the ambient light. This makes the spectrometers equally easy to operate under all ambient lighting conditions. 

There were already some commercial products of reflectance spectrometers based on active light sources since 2000, such as GreenSeeker (from Trimble Navigation) [26], Crop Circle (from Holland Scientific Inc.) [27], CropSpec (from Topcon Precision Agriculture Inc.) [28] and N-sensor ALS serial products (from Yara Inc.) [29]. The working principle of these reflectance spectrometers developed by different companies is basically similar, but they have their own application characteristics and suitable application environments because of differences in detection wavebands, the range of measurement heights, and the arrangement of the light sources (vertical or oblique) [17,21]. GreenSeeker adopts an Light-Emitting Diode (LED) light source with center wavelengths of 660 and 770 nm. The radiation surface of the light source is elliptical, and the effective measurement height range is 60–160 cm. The Crop Circle ACS470 adopts a multi-color LED light source whose central wavelengths can be adjusted by changing optical filters. The multi-channel detectors can sense the reflected light of the target simultaneously. The N-Sensors ALS uses a flashing hernia lamp as the light source, including the left and right detectors to measure the crop canopy on both sides of the vehicle. It can reach a large size of detection area because of very high measurement height. The central wavelengths of Cropspec are 735 and 808 nm, which is from a pulsed laser light source. The light source can be installed 2–4 m high with a Field of View (FOV) of 45–55° to form a large detection area. Generally, handheld spectrometers (such as Greenseeker and CropCircle) tend to be lightweight, with LEDs as light sources, and are mainly used to measure small areas (usually in the range of 0.1–0.5 m^2^) [30]. Reflectance spectrometers for large area detection (such as Cropspec and N-sensors ALS) usually use high-power light sources (modulated laser or hernia lamp for instance) with high measurement heights (over 2 m) to increase the detection area (some are even greater than 10 m^2^).

Manufacturers of spectrometers were reluctant to provide technical material because of commercial benefits. At the same time, few researchers conducted research on the general scientific or engineering issues of such spectrometers. Spectral diagnosis technology depends on the quantitative relationship model between crop growth and specific VIs. In recent years, the increasing application of hyperspectral spectrometers had greatly promoted the establishment of this relationship model. Because of the particularity of the detection wavebands used in the relationship models which were established by different researchers, it is difficult to purchase a ready-made spectrometer to carry out the relevant research. This makes the scientific research outcomes difficult to apply in real production.

In this paper, we developed a reflectance spectrometer based on an active light source with central wavelengths of 730 and 810 nm and provided a method for designing this kind of spectrometer. The VIs in the form of ratio (such as Normalized Difference Vegetation Index (NDVI) and Ratio Vegetation Index (RVI)) can be measured independent of the measurement height and the ambient light environment. The performance tests were carried out in the field to verify the feasibility and reliability of the method. 

## 2. Materials and Methods

In this section, starting from the problems with the conventional calculation method of VIs, a new calculation method for VIs based on an active light source is proposed (an active light source is a kind of artificial light source which is equipped as a part of the spectrometer, and usually produces modulated light in two or more special wavebands), and the measurement conditions that the calculation method should meet are analyzed. Finally, a reflectance spectrometer based on this new method is introduced.

### 2.1. Method for Calculating Vegetation Index

The Normalized Difference Vegetation Index (NDVI) and the Ratio Vegetation Index (RVI) are the most commonly used VIs in the form of ratios, and they reflect the difference in vegetation coverage and growth conditions well, being especially suitable for detecting vegetation with vigorous growth and high coverage. But the conventional calculation method of VIs has many inconveniences and difficulties in practical applications.

#### 2.1.1. Conventional Method for Calculating Vegetation Index

The conventional calculation formula of NDVI and RVI are shown in Equations (1) and (2), respectively.

(1) $$NDVI=\frac{R_{\lambda_{1}}-R_{\lambda_{2}}}{R_{\lambda_{1}}+R_{\lambda_{2}}}$$

(2) $$RVI=\frac{R_{\lambda_{2}}}{R_{\lambda_{1}}}$$

where *R_λ_* is the reflectance of the specific waveband whose central wavelength is λ, *λ*_1_ and *λ*_2_ are central wavelengths of near-infrared and visible light bands for measurement, respectively.

The measurement of the reflectance *R_λ_* in Equations (1) and (2) requires a specific measurement height in the case of active light, which can be expressed as the ratio of canopy to whiteboard-reflected radiation in a specific waveband. 

The formula for calculating reflectance can be described as Equation (3).

(3) $$R_{\lambda }=\frac{L_{C\lambda -H}}{L_{W\lambda -H}}$$

where *L_Wλ-H_* is the response value of the spectrometer to the whiteboard-reflected radiation of the waveband with central wavelength *λ* at the measurement height *H*, *L_Cλ-H_* is the response value of the spectrometer to the canopy-reflected radiation of the waveband with central wavelength *λ* at the measurement height *H*. 

From Equations (1) and (2), it can be known that the calculation of VIs, such as NDVI and RVI, need to obtain the reflectance of the detection light wavebands. Equation (3) shows that the reflection value of the standard whiteboard at a specific height is required as the reference value for the calculation of reflectance. Therefore, the spectrometer is required to be fixed at a certain height in actual use, which makes the actual operation very inconvenient.

#### 2.1.2. Method for Calculating Height-Independent Vegetation Indices

To express the calculation method of VIs in a new form, the standard whiteboard response ratio coefficient *C_W_* is introduced into the equations and defined as Equation (4):

(4) $$C_{W}=\frac{L_{W\lambda_{1}-H}}{L_{W\lambda_{2}-H}}$$

*C_W_* can be measured by experiments. Moreover, the NDVI and RVI can be rephrased as the sum of Equations (5) and (6) with C_W_.

(5) $$NDVI=\frac{L_{C\lambda_{1}-H}-C_{W}\cdot L_{C\lambda_{2}-H}}{L_{C\lambda_{1}-H}+C_{W}\cdot L_{C\lambda_{2}-H}}$$

(6) $$RVI=\frac{L_{C\lambda_{1}-H}}{C_{W}\cdot L_{C\lambda_{2}-H}}$$

For instance, the corresponding derivation steps of Equation (5) can be expressed as follows:

$$\begin{array}{ll} NDVI & =\frac{R_{\lambda_{1}}-R_{\lambda_{2}}}{R_{\lambda_{1}}+R_{\lambda_{2}}}=\frac{\frac{L_{C\lambda_{1}-H}}{L_{W\lambda_{1}-H}}-\frac{L_{C\lambda_{2}-H}}{L_{W\lambda_{2}-H}}}{\frac{L_{C\lambda_{1}-H}}{L_{W\lambda_{1}-H}}+\frac{L_{C\lambda_{2}-H}}{L_{W\lambda_{2}-H}}}\\ & =\frac{\frac{L_{C\lambda_{1}-H}}{C_{W}\cdot L_{W\lambda_{2}-H}}-\frac{L_{C\lambda_{2}-H}}{L_{W\lambda_{2}-H}}}{\frac{L_{C\lambda_{1}-H}}{C_{W}\cdot L_{W\lambda_{2}-H}}+\frac{L_{C\lambda_{2}-H}}{L_{W\lambda_{2}-H}}}\\ & =\frac{L_{C\lambda_{1}-H}-C_{W}\cdot L_{C\lambda_{2}-H}}{L_{C\lambda_{1}-H}+C_{W}\cdot L_{C\lambda_{2}-H}} \end{array}$$

If the value of *C_W_* keeps constant independent of the measuring height, the calculation method, which is expressed by Equations (5) and (6), is fundamentally different from that expressed by Equations (1) and (2). Equations (5) and (6) indicate that NDVI and RVI can be tested independent of the measurement height, which greatly facilitates the practical application of the spectrometers since there is no need to fix the measurement height.

The test results of the reflectance sensor (developed by METER Group, Inc. in Pullman, WA, USA) showed that the ratio of red to NIR spectral irradiance α can be used as a rough approximation at midday under clear sky conditions. But this conclusion was not extensively tested since any fluctuations in α may occur with changes in atmospheric conditions or across large variations in sun elevation angle [31]. The meaning of ratio α is similar to *C_W_* expressed in Equation (4), except that α is the radiation ratio for a natural light environment, while *C_W_* is that of an active light environment. *C_W_* has more stable characteristics than α because the value of *C_W_* is independent of the natural light environment.

To keep the *C_W_* constant with the measurement height changes, two criteria need to be met for designing spectrometers. (i) The two different spectral measurement wavebands of active light source form the same or largely the same irradiation area. (ii) The two photosensitive detectors for different spectral reflectance need to be installed within close proximity to each other, which can ensure basically the same incident angle for each reflecting point to the two photosensitive detectors.

When the functional design of the spectrometer meets the above two criteria, the signal intensity detected by the photosensitive detectors is directly proportional to the intensity of the reflected radiation because of the same FOV. Moreover, the ratio of the radiation intensity of the two spectral wavebands emitted by the active light source is unchanged, which ensures that *C_W_* maintains a constant value.

### 2.2. Design Scheme of Reflection Spectrum Measuring Device

In this section, a design scheme for an active light source and optical detector system is proposed to meet the design criteria proposed in Section 2.1, and the impacts of relevant design parameters on measurement results are analyzed theoretically.

#### 2.2.1. Layout of Active Light Source

**Figure 1:**
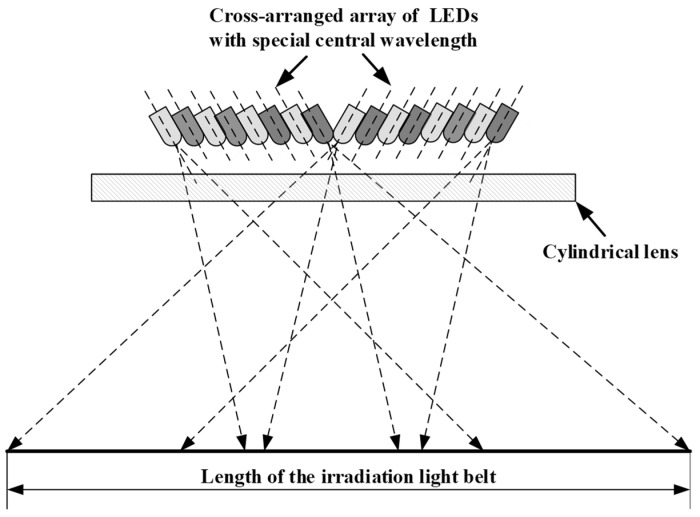
Layout of active light source.

The active light source mainly consists of an array of LEDs with specific central wavelengths and a cylindrical lens. To make the active light source with two different detection wavebands produce a uniform illumination area and make the spectrometer meet the first item of the design criteria, the LEDs with different wavebands in the array are arranged interlaced with each other in the light source layout shown in Figure 1. The central optical axis of the LED is inclined in a vertical, downward direction to expand the illumination area. The emitting light of the LEDs is reshaped by the cylindrical lens and forms an approximate rectangular illumination area.

#### 2.2.2. Parameter Design Requirements for Detecting Light Path System

**Figure 2:**
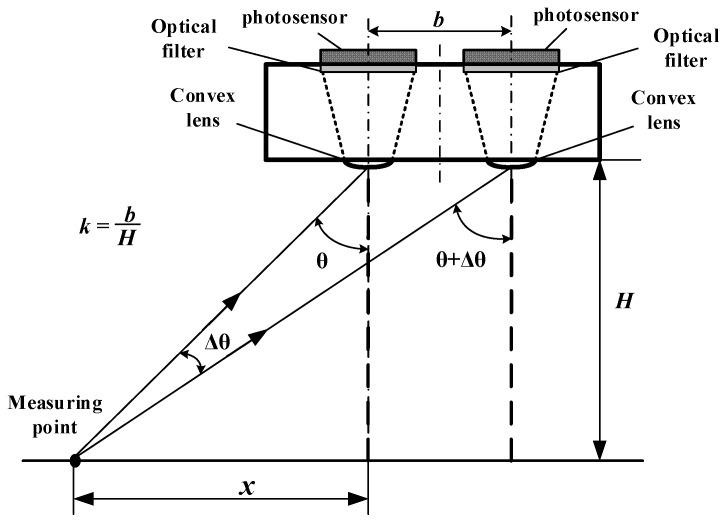
Principle diagram of reflective light detection optical system.

To detect the intensity of reflected light with different wavebands, two optical detection paths are designed. The distance between the central axes of the two optical detection paths is represented by *b*, and the measurement height is expressed by *H*. A schematic diagram of the optical detection paths is shown in Figure 2. 

Because the intensity of the reflected light entering the optical path system from each reflecting point is proportional to the cosine value of the incident angle *θ*, the relative deviation of the reflected light intensity between the two optical detection paths *E* can be expressed by Equation (7).

(7) $$E=\frac{\cos(\theta )-\cos(\theta +\Delta \theta )}{\cos(\theta )}\times 100\%=(1-\frac{\sqrt{H^{2}+x^{2}}}{\sqrt{H^{2}+{\left(x+b\right)}^{2}}})\times 100\%$$

The position *x_max* where *E* reaches its maximum can be calculated with Equation (8) which is obtained from the derivative of Equation (7). Moreover, the maximum value of *E*, expressed by *E_max,* can be calculated with Equation (9):

(8) $$x\_\max=\frac{\sqrt{H^{2}+b^{2}}-b}{2}$$

(9) $$E\_\max=(1-\sqrt{\frac{4+{\left(\sqrt{k^{2}+4}-k\right)}^{2}}{4+{\left(\sqrt{k^{2}+4}+k\right)}^{2}}})\times 100\%$$

where $k=\frac{b}{H}$. From Equation (9), *E* can be controlled within the expected range by selecting appropriate values for parameters *b* and *H*. The design process of determining parameter *k* is actually the process of meeting the design criterion (ii).

### 2.3. Reflectance Spectrometer

In this section, we will introduce a self-developed spectrometer which is a specific-spectral type of spectrometer based on an active light source with 730 and 810 nm as the central wavelengths. The spectrometer only extracts the response information caused by the active light source to calculate the VIs, and the response information from ambient light sources is effectively eliminated.

#### 2.3.1. Working Principle of the Spectrometer

**Figure 3:**
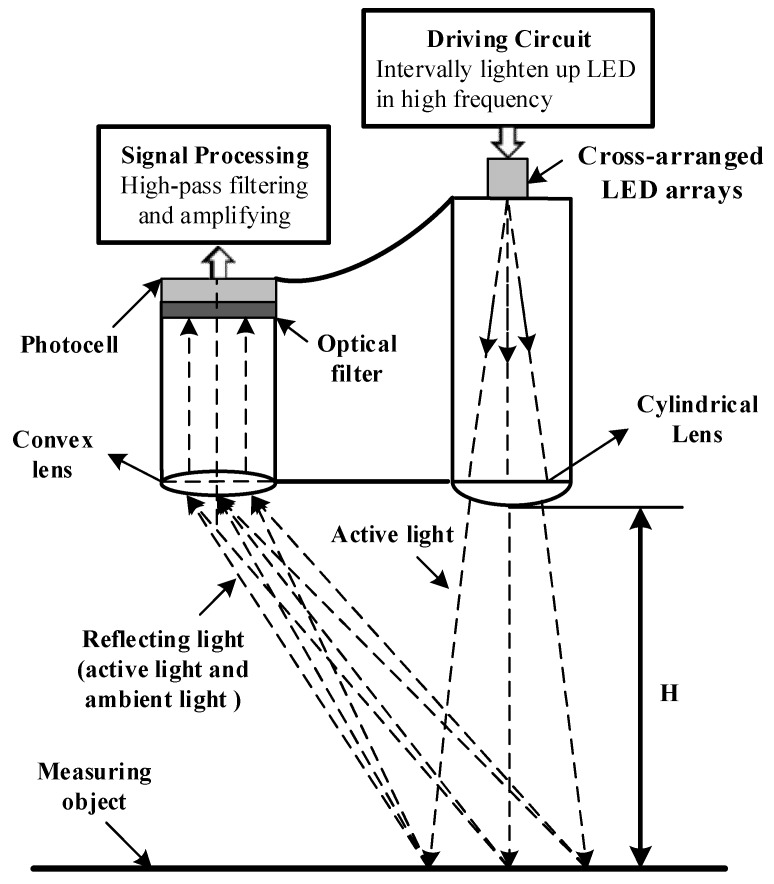
Working principle of dual-band reflectance spectrometer.

The working principle of the dual-band reflectance spectrometer is shown in Figure 3. The LEDs are illuminated in a high frequency interval mode. In this mode of operation, LEDs work for every 15 ms, during which they are illuminated at a frequency of 2000 Hz, and then stop working for 85 ms. This working mode can ensure the LEDs hold a stable central wavelength, because continuous illumination results in the obvious increase in the LEDs’ junction temperature and a red shift in the emission spectrum of LEDs [32,33]. The rectangular illumination area is formed after being reshaped by the cylindrical lens. The photocell is used as a photosensitive detector. The reflected light, including both the active light and the sunlight, is focused by convex lens and filtered by optical filters to form narrow band light with a specific bandwidth and central wavelengths, which is finally received by the photocell. So, the response signal of the photocell is a superposition result of active light and sunlight response signals; the amplitude of the photocell response signal is the sum of the amplitude of the response signal caused by active light and sunlight independently.

**Figure 4:**
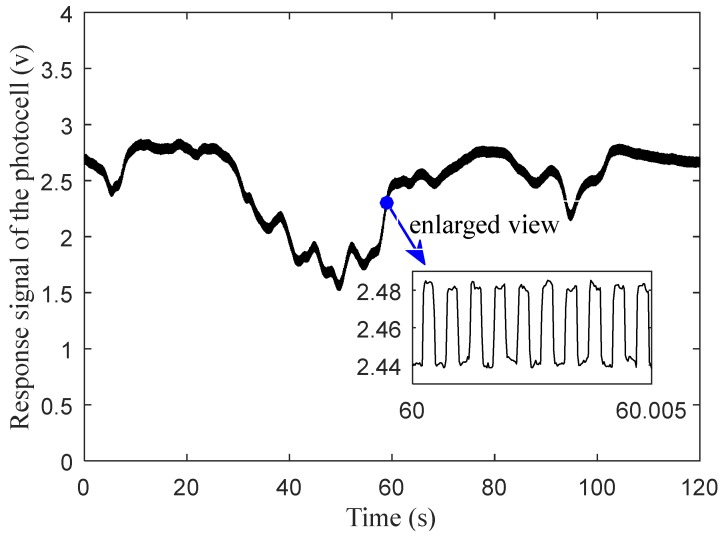
Response signal of the photocell to the superposition of active light and sunlight.

Figure 4 shows the two-minute-long sampling results of the photocell response signal when the spectrometer was fixed in a spot of a wheat field in a sunny day (there were clouds and occasionally breezes during the test.). The amplitude of the response signal caused by active light was very small compared with that caused by the sunlight. So, the amplitude changing trend of the photocell response signal was determined by the response signal caused by sunlight (or ambient light) which changed relatively slowly and was simultaneously superimposed together with the square wave signal at 2000 Hz, which was caused by the active light. Due to the obvious difference in frequency between the two types of response signals, the response signal caused by the active light can be easily extracted from the photocell response signal through high pass filtering and can maintain its own original amplitude, so as to eliminate the influence of sunlight on the measurement results. 

**Figure 5:**
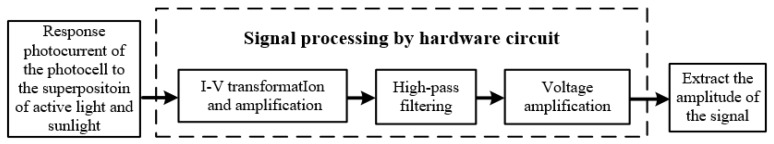
Procedure of processing response signal of the photocell.

**Figure 6:**
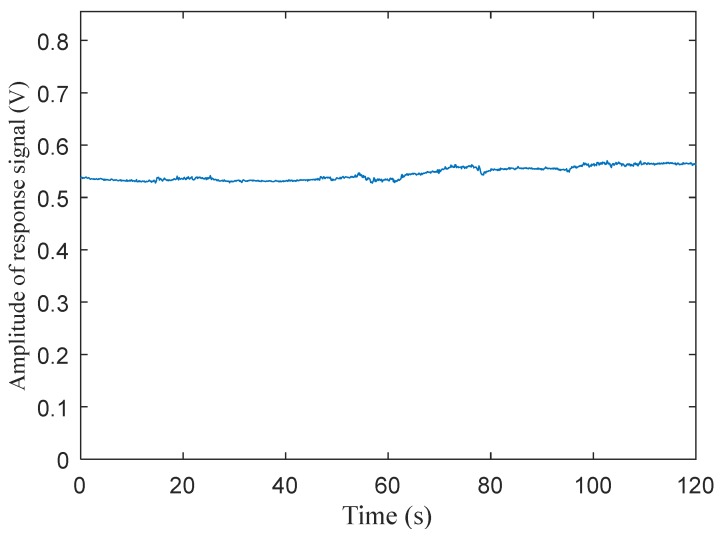
The amplitude of the response signal caused by active light after being separated from the photocell response signal and, secondly, amplified.

The whole signal processing flow is expressed in Figure 5. As shown in the processing procedure shown in Figure 5, the response signal of the photocell, which is illustrated in Figure 4, was firstly filtered by the high-pass filter to separate the response signal caused by active light. Then, the filtered signal, which was a signal with a high frequency square wave, was amplified again to extract the amplitude of the signal. The amplitude of the active light response signal after it was amplified for a second time is shown in Figure 6. The method for how to extract the amplitude of the active light can be found in [33]. Both the average and standard deviation of the amplitude of the response signal, shown in Figure 6, were 0.545 and 0.013, respectively. It was indicated that the amplitude of the response signal caused by active light was stable, though the sunlight intensity kept changing during the test, and the response signal caused by sunlight was efficiently removed from the response of the photocell after being filtered. It should be pointed out that the fluctuation in response amplitude may be caused by circuit interference or disturbance of the crop canopy caused by the wind.

#### 2.3.2. Main Working Elements and Performance Parameters of the Spectrometer

**Figure 7:**
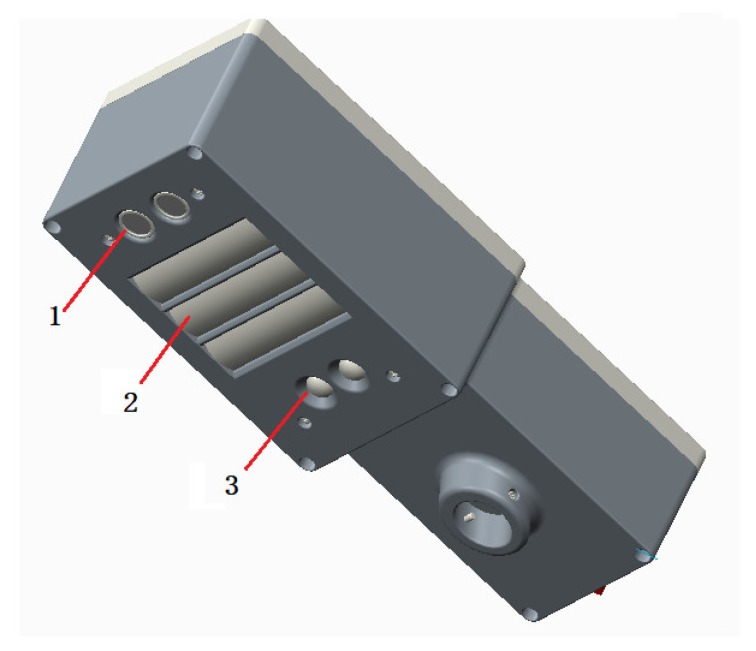
Self-developed dual-band reflectance spectrometer. (1) Ultrasound ranging sensor, (2) narrow band active light source, (3) reflected light detector.

The self-developed reflectance spectrometer according to the above design principles is shown as Figure 7. The main working elements of the spectrometer include LEDs with specific central wavelengths, a cylindrical lens, a convex lens, an optical filter and a photocell. The array of LEDs and the cylindrical lens constitute the active light source; the convex lens and optical filters are the main components of the optical detection paths. 

(1) LEDs and Optical Filters

**Figure 8:**
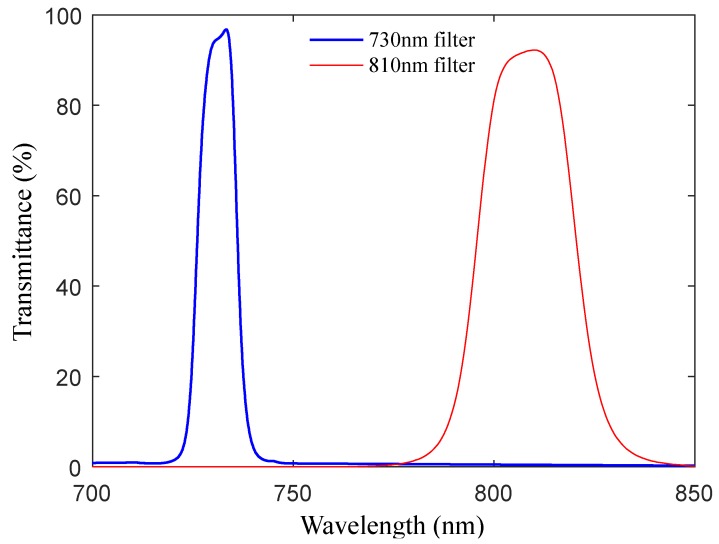
The light transmittance of two different optical filters.

**Figure 9:**
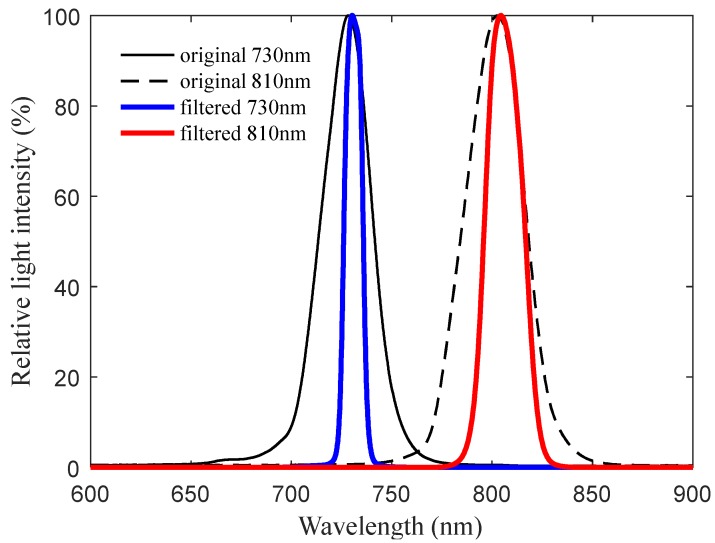
The relative light intensity distribution of Light-Emitting Diode (LED) emission light before and after being filtered by the optical filter.

The central wavelengths of the reflectance spectrometer are 730 and 810 nm, and the full width at half maximum (FWHM) is less than 20nm. The optical filters (by Beijing Xintian Borui Photoelectric Technology Co., Ltd., Beijing, China) are covered on the surface of the photocells to further reduce the bandwidth of the reflected active light since the original FWHM of the LEDs (XL302440NRC-730 and XL302430HIRC-810, by Nanning Lvxing Lighting Electronics Co., Ltd., Nanning, China) cannot meet the design requirement. The light transmittance of two different optical filters is shown in Figure 8, and the relative light intensity distribution of LED emission light before and after passing through the optical filters is shown in Figure 9 (the testing data of the LEDs and filters are all provided by the manufacturers). The final central wavelengths of the 730 and 810 nm wavebands are 730.3 and 809.5 nm, respectively, and the FWHM values are 10.8 and 19.5 nm.

(2) Cylindrical Lens and Convex Lens

The size of the single cylindrical lens is 80 mm × 20 mm, and its focal length is 30 mm. The LED arrays are installed where the focal line of the cylindrical lens is located (26.14 mm away from the plane of the cylindrical lens). With the action of the cylindrical lens, the active light source forms a more concentrated irradiation area, which improves the detectability of the reflected light.

The focal length of the convex lens is 15 mm, and the distance between the photocell surface and the plane of the convex lens is 10.8 mm where the focus plane of the convex lens is located. The focusing function of the convex lens ensures that all the light reflected into the spectrometer falls into the detection area of the photocell. The two lenses are made of BK7 glass, which is the product of the Shotto Glass Company in Germany.

(3) Photocells

**Figure 10:**
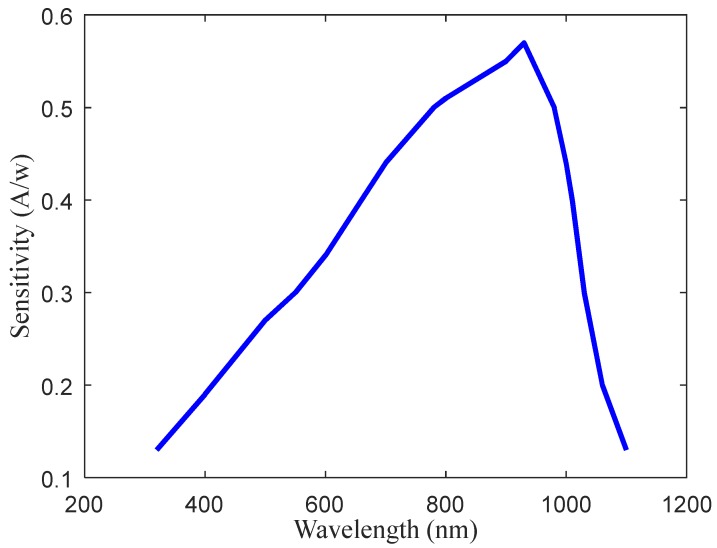
The sensitivity of the photocell.

A type of silicon photocell (2CR40, by Shanghai Qinyue Electronic Technology Co., Ltd., Shanghai, China) with high sensitivity and wide spectral wavebands is used as the detector for reflected light radiance, its detection area is 10 mm × 20 mm, and its sensitivity curve is shown in Figure 10, which shows its higher sensitivity at wavelengths of 730 and 810 nm (sensitivity data are provided by the manufacturer). The typical response time of the photocell is 70 μs which can match the requirement for detecting signals with a high frequency of 2000 Hz.

(4) Working Parameters of the Spectrometer

**Figure 11:**
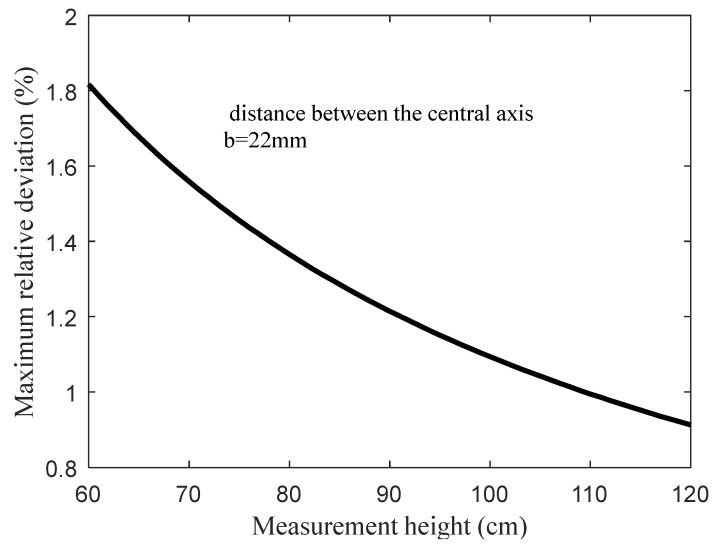
The maximum relative deviation of incident light intensity between the two optical detection paths when the measurement height changes.

The working frequency of the LEDs set in the spectrometer is 2000 Hz. After being reshaped by the cylindrical lens, the irradiation of the active light forms an approximate rectangular area whose geometric dimension meets the following relationship: width = 0.25× height, length = height. The distance between the central axes of the two optical detection paths *b* = 22 mm (see Figure 2), and the designed effective range of measurement height of the spectrometer is 60–120 cm. When the measurement height changes, the theoretical maximum relative intensity deviation of the incident light entering the two optical detection paths is shown in Figure 11 (the theoretical maximum relative intensity deviation can be calculated with Equation (9) in Section 2.2.2). It can be seen from Figure 11 that the maximum relative deviation of the incident light intensity is less than 2% within the effective range of the measurement height.

### 2.4. Calibration of the Spectrometer

To evaluate the stability and accuracy of the spectrometer, some standard grayscale plates (Hefei Xingyue Luminous Technology Applications Institute, Hefei, China) were used as the measurement objects to carry out the calibration tests. The reflectance measured by the spectrometer was compared with the nominal reflectance of the grayscale plates to evaluate the accuracy of the spectrometer. On other hand, the standard whiteboard response ratio coefficient *C_W_* was tested in different measurement height in indoor and outdoor environments to value the stability of the spectrometer.

#### 2.4.1. Reflectance Measurement to Standard Grayscale Plates

Six standard grayscale plates were used as the test objects, whose nominal reflectances are 5%, 10%, 20%, 40%, 60% and 75%, respectively. Moreover, the standard whiteboard (made by the same producer as the grayscale board) with a nominal reflectance of 99%, was treated as the reference object. The size of all the grayscale plates and the whiteboard is 60 mm × 60 cm. The spectrometer was placed 70 cm above the grayscale plate during the tests. The experiments were carried out in an indoor laboratory environment under fluorescent lamps. To eliminate the reflection interference outside the grayscale plate as much as possible, a black cotton cloth was placed under the grayscale board. The reflectance of the black cotton cloth was less than 2%. 

**Figure 12:**
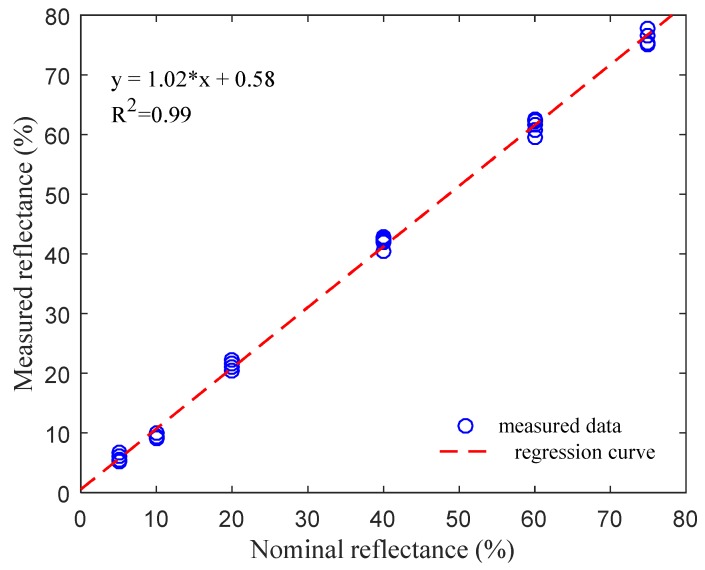
Linear regression results of measured and nominal values for 730 and 810 nm wavebands.

Once the response of the spectrometer to each grayscale plate and whiteboard were measured, the reflectance of each grayscale plate was calculated with Equation (3). The reflectance of each grayscale plate was measured three times during tests. Figure 12 shows the linear regression results based on the measured reflectance (both at 730 and 810 nm) and the nominal reflectance of the grayscale plates. The linear regression results showed that the designed spectrometer had stable and accurate measurement results.

#### 2.4.2. Determination of the Standard Whiteboard Response Ratio Coefficient *C_W_*

**Figure 13:**
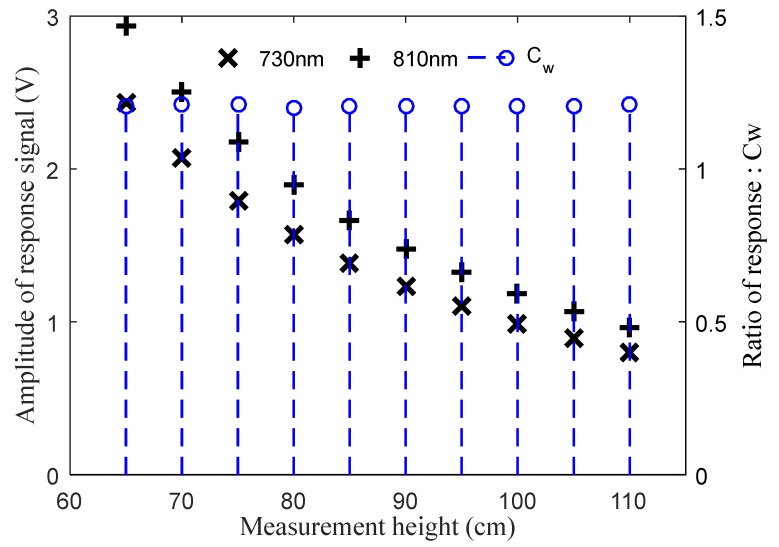
The measured value of C_W_ indoors at different measurement heights.

Ten measurement heights, at an interval of 5 cm, between 65 and 110 cm were set during indoor tests. The test process at each measurement height lasted for 30 s, during which the data update frequency was 10 Hz. The average value of those recorded data in 30 seconds was finally taken as the whiteboard response value of the spectrometer to calculate the *C_W_*. Figure 13 shows the reflection response of the spectrometer to the standard whiteboard and the *C_W_* at different measurement heights.

**Figure 14:**
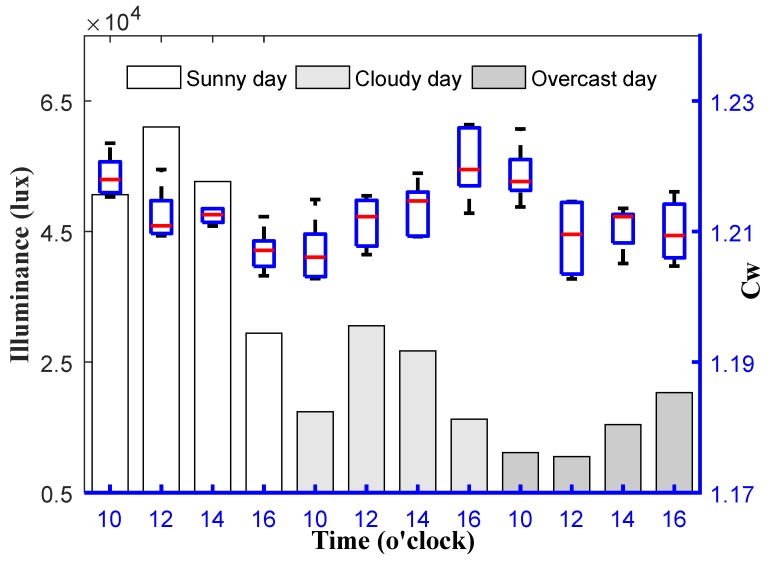
The measured value of C_W_ outdoors at different measurement heights under different weather conditions.

Three days in different weather conditions (sunny, cloudy and overcast) were selected to carry out the outdoor tests. Four testing time points of 10:00, 12:00, 14:00 and 16:00 were selected to start the repeated tests each day, during which the *C_W_* were measured at five heights (at an interval of 10 cm, from 60 to 100 cm). Each test (including the five measurement heights) lasted about 15 min. The minimum and maximum illuminance of ambient light were recorded by two digital illuminance meters (AS823, by Smart Sensor^®^, Shenzhen, China) during each test. The test value of *C_W_* at different measurement heights at each testing time point are shown as a boxplot in Figure 14. The average illuminance at each testing time point outdoors is displayed as the bars in Figure 14 for reference. The illuminance conditions during each test outdoors are described in Table 1.

The mean value of *C_W_* in an indoor environment was 1.206, the standard deviation was 0.0034, and the coefficient of variation (CV) of *C_W_* was 0.26%. The corresponding results of *C_W_* outdoors under different weather conditions were 1.213, 0.0058 and 0.48%, respectively. It indicated that *C_W_* kept a high stability when the measurement height changed and kept constant whether indoors or outdoors. The results of the outdoor tests were highly consistent with those of the indoor tests. This provided a guarantee for the reliable measurement of field vegetation indices.

## 3. Field tests, Results and Discussion

### 3.1. Test Condition and Test Design

The field performance tests of the spectrometer were carried out in a wheat experimental area of Nanjing Agricultural University on 12 April 2019, and the wheat (during the experimental tests) was in the jointing stage. In particular, the active light source of the spectrometer was illuminated in a high frequency interval mode (2000 Hz). Unlike the passive light spectrometer, we did not need to carry out our tests in a specific period of time, we took some tests in the morning, after 10:00, and the rest of the tests in the afternoon, after 15:00. The field test scheme was shown in Table 2.

**Figure 15:**
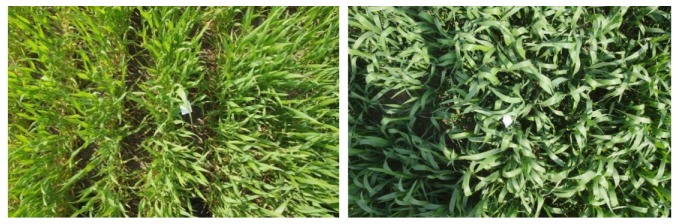
Photographs of wheat growth under different nitrogen application rate levels. (left: 150 kg/ha, right: 300 kg/ha).

There were two nitrogen application rate levels (NARLs) in the experimental fields, 150 and 300 kg/ha. The size of a single experimental field was 5 m × 7 m. Four test fields were selected for each NARL, five test points were selected in each field, and five measurement heights were set for each test point. The measurement height was set from 65 to 105 cm with a 10 cm interval. In order to eliminate the influence of soil background interference on the measurement results, the test points were selected in an area where the wheat canopy basically covered the soil background and the crop growth was relatively uniform. The growth of wheat seedlings at the test points is shown in Figure 15.

### 3.2. Experimental Results

The NDVI and RVI during tests were defined as:

(10) $$NDVI=\frac{R_{810}-R_{730}}{R_{810}+R_{730}}\;\mathrm{and}\;RVI=\frac{R_{730}}{R_{810}}$$

**Figure 16:**
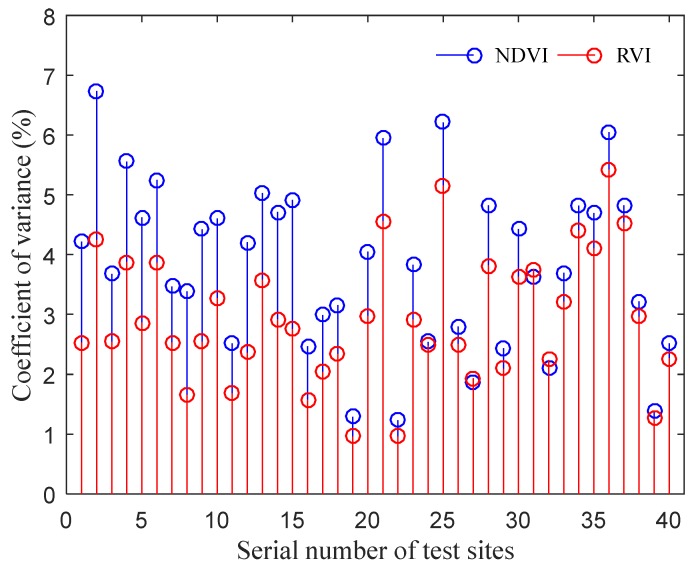
The coefficient of variation (CV) of the statistical results for Normalized Difference Vegetation Index (NDVI) and Ratio Vegetation Index (RVI) in each test point.

The CV of the NDVI and RVI at each test point is shown in Figure 16. The statistical results in Figure 16 were calculated by taking the data at all the measurement heights of each test point and treating them as an analysis unit. The mean and standard deviation of CV for the NDVI were 3.85% and 1.39% respectively, the corresponding statistical results for RVI were 2.93% and 1.09%. 

Because a change in the measurement height causes a change in the FOV of the spectrometer, in order to analyze the impact of this change on the measurement results, we analyzed the CV of the measurement results between the adjacent heights in each test point (the distance between each adjacent height was 10 cm, and each test point contained four adjacent measurement data). The mean and standard deviation of CV for NDVI were 1.75% and 1.46%, respectively, and the corresponding statistical results for RVI were 1.29% and 1.05%. These data show that the smaller the height difference is, the smaller the CV is.

### 3.3. Analysis of Variance

The fertility of the test fields (mainly depending on NARL) and the measurement height of the spectrometer are the two main factors that may affect the measurement results of the vegetation indices. Therefore, we designed a two-way ANOVA to test whether the two factors have a significant impact on the measured VIs. We divided the measurement height into five levels, corresponding to the five measurement heights during field tests, and divided the NARL into two levels, coinciding with the actual N fertilization treatments of the tests. In order to comprehensively analyze the test data, we took all the test data as the sample data of ANOVA and considered the test data of each of the five test points in each test field block as one group of experimental data. Thus, a total of 40 groups of data were obtained for ANOVA, including 20 groups of lower NARL and 20 groups of higher NARL. According to this design, two-way ANOVA for NDVI and RVI was carried out. The results of the two-way ANOVA are shown in Table 3. The results show that the measurement height had no significant effect on the measurement results of VIs, but there were significant differences among the data from the field blocks with different NARLs.

**Figure 17:**
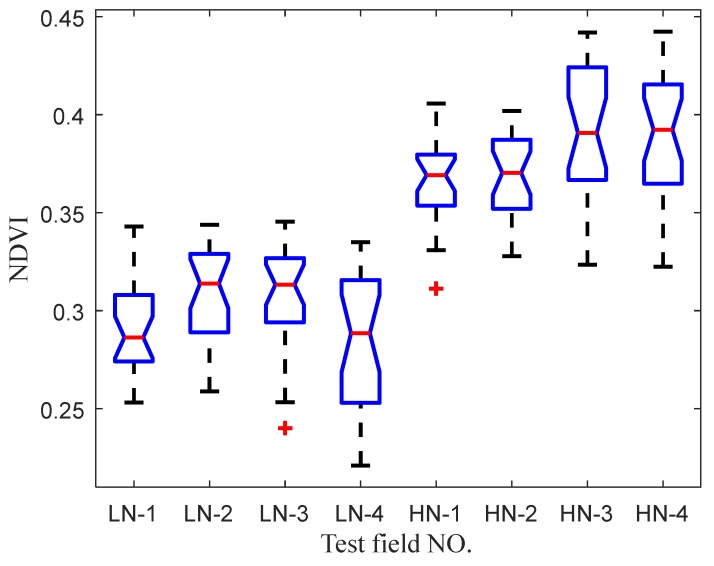
Boxplot of NDVI values in test fields with different nitrogen application rate levels.

**Figure 18:**
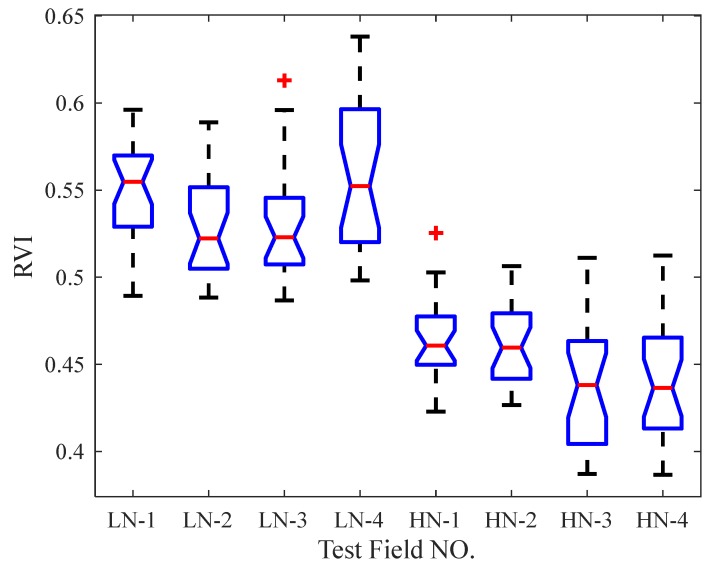
Boxplot of RVI values in test fields with different nitrogen application rate levels.

In order to further analyze whether this significant difference results from the difference in NARL, we conducted a one-way ANOVA for NARL. Based on the results of the one-way ANOVA, we introduced multiple comparative tests (adopting Tukey’s Honestly Significant Difference Procedure) to determine the cause of the significant difference of VIs by pairwise comparison analysis [34]. During one-way ANOVA, all test points data of each test field block were taken as one group of experimental data (with 25 results in each group) to finally form a total of eight groups of data. According to the different NARLs, the field blocks with higher N fertilization treatment were labeled as HN-1–HN-4 respectively and, correspondingly, the field blocks with lower N fertilization treatment were labeled as LN-1–LN-4. The boxplots of NDVI and RVI measurement values for one-way ANOVA are shown in Figure 17 and Figure 18, respectively. The results of the multiple comparison tests are described in Table 4. According to the comparisons between the confidence intervals of NDVI and RVI values in each test field block, it can be found that there was a significant difference in the test data between any two groups with different NARLs, while no significant difference existed between any two groups with the same NARL, except for the RVI test groups LN-2 and LN-4.

### 3.4. Discussion

The reflectance measurement described in Section 2.4.1 indicates the high accuracy of the spectrometer. So, the reliability, stability and deviations in the measurement results are the biggest issues in the field tests. The following is an analysis of these issues.

(1) We can find two sets of data with obviously different distributions, corresponding to two different NARLs (Figure 17 and Figure 18). Moreover, the analysis of the multiple comparison tests showed that differences in the test data were the result of different NARLs. The results of ANOVA and the multiple comparison tests specifically expressed a significant correlation between the NARLs and VIs. The NDVI had a positive response to the NARL, while the RVI negatively responded to the NARL. This shows that neither NDVI nor RVI measured by the spectrometer had the ability to discriminate the NARLs in the field. This indicates the reasonability of the measurement results of the spectrometer;
(2) The results of the ANOVA indicated that the measurement height had no significant influence on VIs. Furthermore, this indirectly expressed that there were no obvious differences in the measurement values of VIs by the spectrometer when the measurement height changed. The results from Figure 16 show that there were no significant differences in the CV values of VIs under different nitrogen application rate levels, and the CV distribution of NDVI and RVI data at all test points was similar. The measured results of VIs kept high stability as the measurement height changed, which proved that the measurement characteristics of the spectrometer were consistent with the designexpectations;
(3) Compared with the stability of *C_W_* in Section 2.4, the stability of the measured VIs in the field was significantly lower. This was due to the fact that the measured object was a whiteboard and the surface properties of the object were consistent during the determining tests of C_W_. The characteristics of the measured objects (canopy) changed with the measurement height because of the variation in FOV of the spectrometer. This affected the stability of the measurement results in actual field tests. During the experiments, the maximum difference in the measurement height at the same test point was 40 cm, and the maximum relative deviation of the radiation area of the active light source theoretically reached 61.6% (based on the geometric characteristics of the irradiation area, which are described in Section 2.3.2, part (4)). The statistical results of the CV between the adjacent heights in each test point were significantly better than those shown in Figure 16, because the relative deviation of the canopy characteristics was smaller between the adjacent heights. So, the measurement deviation at different heights may be largely affected by a change in the measuring object.

## 4. Conclusion

Some researches had shown that the spectral irradiance ratio of different wavebands can be used as a rough approximation during midday under clear sky conditions [31]. Using this characteristic, the structure of the reflectance spectrometer can be simplified. However, due to the changes in the atmospheric conditions and the solar altitude angle, the ratio may have a large fluctuation, which results in poor reliability in practical applications. This is why the performance of the spectrometer based on a passive light source can be affected by the ambient light environment.

This paper provided a method for using an active light source to keep the ratio of reflected irradiance from a standard whiteboard between expected wavebands constant. We introduced the standard whiteboard response ratio coefficient *C_W_* to embody this characteristic. The VIs can be calculated independent of measurement height and ambient light environment based on *C_W_*. This effectively overcomes the defects of passive light source spectrometers. 

Following the design criteria of the active light source proposed in this paper, the C_W_ of the self-developed spectrometer showed a good characteristic of keeping constant at different measurement heights and under indoor or outdoor light environments. The field tests results showed that the measured value of VIs under different measurement heights maintained a high stability, and that the better the consistency of the measured objects inside the effective radiation area of the active light source, the higher the stability of the VI results. The experimental results verified the feasibility and validity of the proposed measurement method and the design criteria. 

However, the current measurement method cannot eliminate the interference caused by soil-reflected irradiance, and the spectrometer is not suitable for large areas of bare soil because the VIs are sensitive to the optical properties of the soil background. For a given amount of vegetation, darker soil substrates result in higher vegetation index values [35]. This is a problem for us and further research is necessary, both by us and by the manufacturers of commercial spectrometers, in order to solve it.

## Figures and Tables

**Table 1:** Illuminance (lux) variation range of ambient light during tests.

	10:00–10:15	12:00–12:15	14:00–14:15	16:00–16:15
**Sunny day on 18 February**	47,650–53,660	59,960–62,130	50,220–55,160	27,960–30,870
**Cloudy day on 19 February**	16,960–17,800	29,430–31,720	25,780–27,670	13,510–19,030
**Overcast day on 21 February**	9586–12,690	9445–11,580	15,010–15,860	19,560–21,090

**Table 2:** Field test scheme.

NARL (kg/ha)	Measurement Height (cm)	Amount of Test Points	Measurement Targets
150	65–105 with a 10 cm interval	20	NDVI, RVI
300	65–105 with a 10 cm interval	20	NDVI, RVI

**Table 3:** Two-way ANOVA results for NDVI and RVI.

Factors	NDVI		RVI	
	F	P	F	P
**NARL**	372.96	0	364.52	0
**MH**	0.37	0.8333	0.29	0.8833
**Interaction**	1.66	0.1599	1.52	0.1965

Note: measurement height (MH).

**Table 4:** Results of multiple comparison tests for NDVI and RVI.

NDVI			RVI		
In LN groups	In HN groups	Between LN and HN groups	In LN groups	In HN groups	Between LN and HN groups
No significant difference	No significant difference	Significant difference	Only LN-2 and LN-4 are significantly different (*p* = 0.0292).	No significant difference	Significant difference
